# A standardized methodical approach to characterize the influence of key parameters on the *in vitro* efficacy of extracorporeal photopheresis

**DOI:** 10.1371/journal.pone.0212835

**Published:** 2019-03-01

**Authors:** Marie Laulhé, Sylvie Lefebvre, Delphine Le Broc-Ryckewaert, Maxime Pierre, Aurélie Ferry, Bruno Delorme

**Affiliations:** MacoPharma, Biotherapy Division, Rue Lorthiois, Mouvaux, France; University of Kentucky, UNITED STATES

## Abstract

Extracorporeal photopheresis (ECP) is an autologous immunomodulatory cell therapy that consists of the *ex vivo* collection of mononuclear cells (MNCs), which are irradiated with UVA in the presence of the photosensitizing agent 8-methoxypsoralen (8-MOP) to induce cell apoptosis. This photoactivated cell preparation is then reinfused into the patient. While the clinical benefits of ECP are well-demonstrated, no study has yet characterized the influence of variations in the composition of the cell preparation on the efficacy of ECP *in vitro*. Here, we describe a standardized methodology for the *in vitro* assessment of ECP that uses the human lymphoma T-cell line and mimics the clinical procedure. By quantifying cell apoptosis, inhibition of cell proliferation, and 8-MOP consumption, we used this approach to characterize the specific influence of key variables on the cellular response to ECP. We found that (i) increases in hematocrit and plasma concentrations attenuated the cellular response to ECP; (ii) plasma concentration was the only variable tested that influenced 8-MOP consumption; and (iii) the loss of efficacy due to variations in the concentration of certain blood components could be counteracted by modulating the UVA dose. This methodology may enable evaluation of other leukapheresis preparation protocols and better determination of the optimal working parameters for ECP.

## Introduction

Extracorporeal photopheresis (ECP) is an immunomodulatory cell therapy in which patients receive autologous infusions of apoptotic mononuclear cells (MNCs) for the treatment of a variety of diseases involving immune dysfunction [1–3]. Although effective and used for several decades, the precise *in vivo* mode of action of ECP is poorly understood [2, 4, 5]. ECP is carried out in 3 stages: 1) MNCs are withdrawn from the patient by apheresis; 2) the cells are then incubated with a photosensitizing agent (8-methoxypsoralen or 8-MOP) and exposed to UVA; and 3) irradiated MNCs are reinfused into the patient [6–8]. 8-MOP passively enters the cells where it intercalates into the DNA within 2 minutes [9, 10]. Upon irradiation with UVA photons, 8-MOP induces DNA cross-linking, which interferes with the cell replication machinery [11–14]. ECP treatment inhibits cell proliferation and induces T-cell apoptosis and the release of immunomodulatory cytokines [6–8]. Irradiation-activated dendritic cells (DCs) transform into antigen-presenting cells (APCs), which recognize and phagocytose ECP-treated apoptotic T-cells and ultimately induce regulatory T-cell (Treg) activation. In graft-versus-host disease (GvHD), Tregs attenuate immune reactions by secreting tolerogenic cytokines [15, 16], while in cutaneous T-cell lymphoma (CTCL), ECP-induced Tregs secrete inflammatory cytokines, which exert immunostimulatory effects on neoplastic cells [6, 17, 18]. Two ECP techniques are currently used to treat immunological disorders: “on-line” and “off-line” ECP, depending on whether MNCs are collected and irradiated using 1 or 2 devices, respectively.

8-MOP and saline solution are added to a leukapheresis preparation composed of plasma, red blood cells, MNCs, and ACD-A (anticoagulant citrate dextrose solution, Solution A) solution, and the mixture is exposed to UVA. The relative proportions of each blood component vary depending on the patient, their pathology, and the treatment protocol [19]. Two different 8-MOP concentrations are routinely used in clinical practice: 200 ng/ml or 333 ng/ml [20–29]. Because the composition of the cell preparation in the irradiated container varies across patients, it is difficult to accurately predict the efficacy and reproducibility of the cellular response. Therefore, this variability should be taken into account in any analysis of the efficacy and limitations of ECP. To our knowledge, no study has investigated the link between the relative proportions of the different components of the cell preparation and the *in vitro* efficacy of ECP.

The aim of our study was to develop a standardized *in vitro* method to systematically quantify the influence on ECP efficacy of each component of the cell preparation and of different UVA doses. Based on a review of the literature and the values used in clinical settings, the following baseline values were established for each parameter: cell density, 300 × 10^6^ cells [19, 30]; ACD/A ratio, 1:14 [31]; 2% hematocrit (HCT) [19, 32]; plasma concentration, 50% v/v in saline [19]; final volume of irradiation container, 300 ml; 8-MOP concentration, 333 ng/ml [25]; UVA dose, 2 J/cm^2^ [32–34]. Experiments were performed by modifying one of the aforementioned parameters at a time. To avoid PBMC donor-related variability we used the JURKAT T-cell line, which has been previously used as a model to determine the *in vitro* efficacy of alternatives to 8-MOP for use in ECP [35, 36]. Advantages of this cell line include (i) cell homogeneity, which improves repeatability between experiments; (ii) composition of pathological T-cell from a lymphoma [37]; (iii) spontaneous proliferation with a constant doubling time [38]; and (iv) apoptosis kinetics similar to those of PBMCs, with a progressive increase in apoptosis 1 and 2 days after ECP treatment [35]. Two endpoints were used to assess ECP efficacy and characterize *in vitro* cellular kinetics after ECP treatment: inhibition of proliferation after 3 days in culture; and apoptosis after 1 and 2 days in culture. Jacob *et al*. proposed that 70% inhibition of proliferation after 3 days of culture post-irradiation can be used as a benchmark to validate ECP treatment efficacy [39], while Taverna and coworkers proposed Δapoptosis measured after 1 day of culture post-irradiation as an earlier measure of ECP quality control [27]. In addition, we measured the residual 8-MOP concentration in the irradiation container after ECP to quantify its consumption.

Our findings show that the composition of the cell preparation affects *in vitro* ECP efficacy. Variations in the HCT and/or plasma ratio reveal a dose-dependent effect for these parameters. However, we show that the system can be adjusted to counterbalance any resulting efficacy loss. Finally, we demonstrate a correlation between proliferation inhibition and Δapoptosis that allow prediction of *in vitro* ECP efficacy as early as 1 day post-irradiation.

## Materials and methods

### - Cell culture

JURKAT cells (Sigma Aldrich, Saint Louis, MO, USA) were cultured at 37°C and 5% CO_2_ in a humidified incubator at a density of 0.2–0.5 × 10^6^ cells/ml in RPMI-1640 medium containing 20 mM HEPES buffer (Life Technologies, Courtaboeuf, France), 2 mM L-glutamine (Lonza, Levallois Perret, France), 100 U/ml penicillin, 100 μg/ml streptomycin (Lonza, Levallois Perret, France), and 10% heat-inactivated fetal bovine serum (Life Technologies, Courtaboeuf, France).

### - ECP procedures and cell isolation

ECP experiments were conducted using the following materials (Maco Pharma, Tourcoing, France): a CE-marked UVA irradiation container, 8-MOP, and a CE-marked MacoGenic G2 UVA irradiation device with a wavelength range of 315–400 nm. The following were added to the irradiation container: ACD-A solution (1/14 v/v) (Maco Pharma), 300 × 10^6^ JURKAT cells (Sigma-Aldrich, Saint Louis, MO, USA) red blood cells (French blood establishment (EFS), Lille, France) to obtain 2% HCT, and a mixture consisting of 50% plasma (EFS, Lille, France) and 50% 0.9% NaCl solution (Maco Pharma), for a final volume of 300 ml. Finally, 8-MOP (333 ng/ml) was added in the irradiation container. For each experiment, a non-irradiated and non-agitated control solution (UVA -) consisting of the same components was used. Plasma from at least 3 donors was pooled for each experiment. UVA irradiation was performed using a MacoGenic G2 set at 2 J/cm^2^. The HCT was verified using an ABX Micros ES60 Hematology Analyzer (Horiba, Kyoto, Japan). After irradiation, JURKAT cells were isolated using a Ficoll/Paque^TM^ density gradient (GE Healthcare Life Sciences, Pittsburgh, PA, USA) and were subsequently washed and cultured as described above.

### - Assessment of apoptosis

Untreated and treated JURKAT cells were incubated at 37°C and 5% CO_2_ for 2 days. After irradiation, cells were double stained with Annexin-V-FITC and propidium iodide (PI) (BD Pharmingen^TM^, San Diego, CA, USA), according to the manufacturer’s instructions, for daily assessment of apoptosis. Samples were analyzed using BD Accuri C6 software (BD Biosciences, San Jose, CA, USA).

The difference in the percentage of Annexin-V-positive cells between untreated and treated cells was determined daily as follows: Δapoptosis = (% Annexin-V-positive cells)_treated_—(% Annexin-V-positive cells)_untreated_.

### - Proliferation assay

Untreated and treated JURKAT cells were incubated at 37°C and 5% CO_2_ for 3 days at an initial density of 0.2 × 10^6^ cells/ml (5 × 10^6^ cells). The total number of cells was determined by automated cell counting (ViCell XR; Beckmann Coulter, Villepinte, France) on day 3 after irradiation.

For each condition tested, the fold increase (FI) in cell proliferation was calculated as follows: FI = total number of cells measured on day 3/ number of cells seeded on day 0.

Cell proliferation inhibition percentage was calculated as follows: proliferation inhibition percentage = 100 × (1-[FI_treated_/FI_untreated_]).

### - Measurement of 8-MOP concentration

Samples were taken from the irradiation container before and after irradiation to measure 8-MOP concentration by high-pressure liquid chromatography (HPLC) (Shimadzu, Noisiel, France). The supernatant was diluted (50/50 v/v) with acetonitrile (VWR, Radnor, PA, USA), vortexed, incubated in darkness, and centrifuged for 15 min at 3,500 rpm. After filtration through a 0.20-μm PTFE filter (Phenomenex, Torrance, CA, USA), the supernatant was analyzed using a 2.3-μm C18 column (internal diameter, 4.6 × 100 mm) and a UV detector set at 296 nm (Shimadzu, Noisiel, France). Fractions were eluted over 25 min using a 0–80% v/v acetonitrile gradient at a constant flow rate of 1.5 ml/min.

### - Statistical analysis

Results are presented as the mean ± standard error of the mean of at least 3 separate experiments performed in duplicate. Differences between conditions were analyzed by one-way ANOVA, and pairwise comparisons were performed using Bonferroni’s multiple comparison test (GraphPad Software, San Diego, CA, USA). A p-value <0.05 was considered significant.

## Results

### - Plasma ratio has a moderate influence on *in vitro* cellular response after irradiation

**Fig 1:**
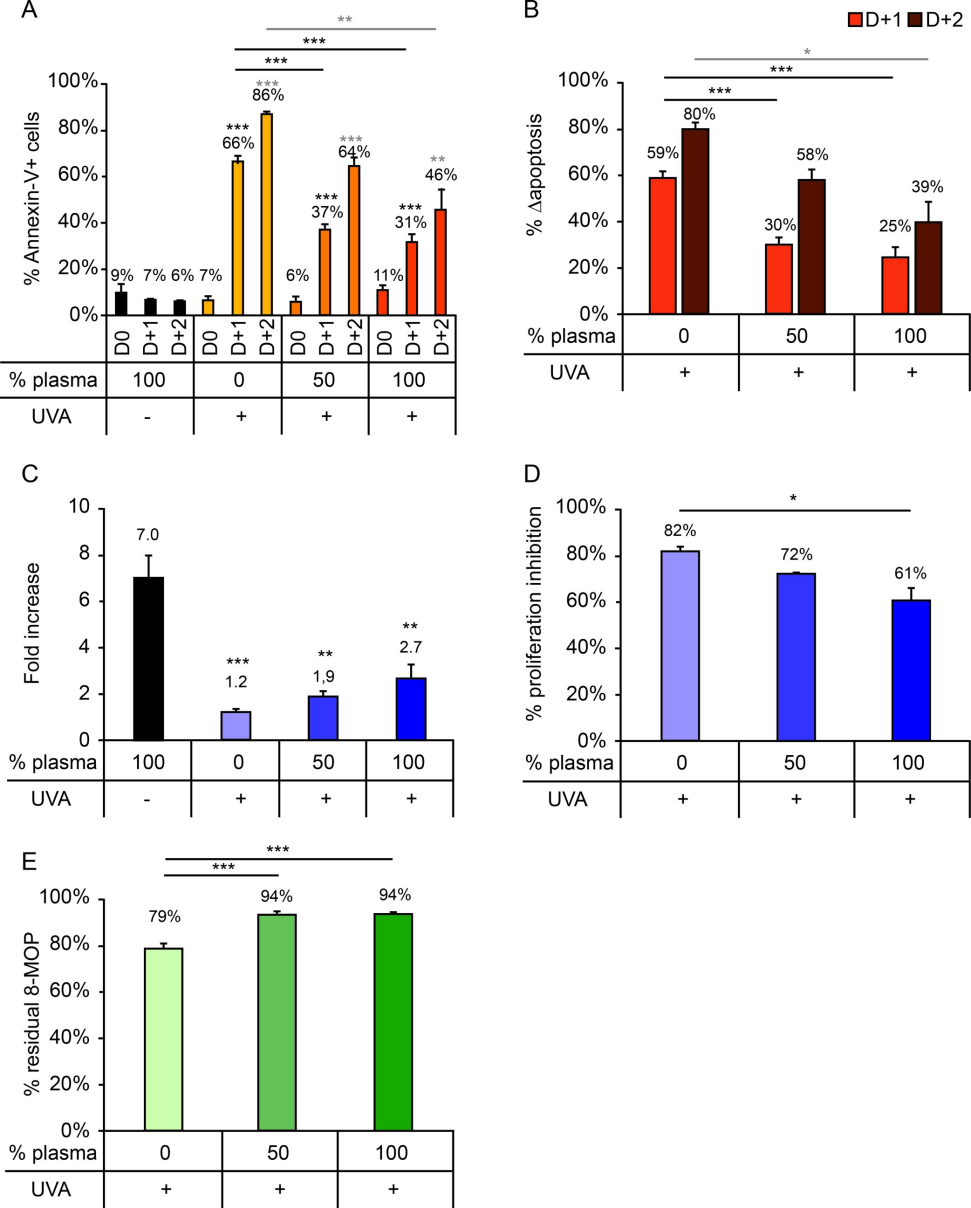
Impact of plasma ratio (0%, 50%, or 100%) on cellular response after UVA irradiation (2 J/cm^2^). (A) Percentage of Annexin-V+ cells measured daily up to 2 days after irradiation. (B) Δapoptosis determined on days 1 and 2 post-irradiation. (C) Fold increase on day 3 post-irradiation relative to day 0. (D) Proliferation inhibition percentage, calculated as the percentage decrease in proliferation in irradiated versus control samples. (E) Residual 8-MOP remaining in the irradiation container after treatment, expressed as a percentage of the pre-irradiation 8-MOP concentration. Pairwise comparisons of each treatment condition were performed. For clarity, only statistically significant results are shown: *p<0.05; **p<0.01; ***p<0.001. Significant differences with respect to the control (UVA -) are indicated with an asterisk above the relevant bar, while comparisons between irradiated samples are indicated with an asterisk over a line linking the relevant bars. For Annexin-V+ cells (A) and Δapoptosis (B), comparisons performed on days 1 and 2 post-irradiation are indicated in black and gray, respectively.

Samples containing 3 different plasma ratio (0%, 50%, and 100%) were tested (Fig 1). For all 3 conditions, we observed a progressive increase in cell death 1 and 2 days after irradiation that was significantly greater than that observed in the non-irradiated control (Fig 1A). No significant differences in apoptosis or Δapoptosis were observed for the 50% or 100% plasma conditions, indicating that the ratio of plasma usually present in the irradiation container during routine ECP procedures has minimal impact on the cellular response (Fig 1A and 1B). However, for the 0% plasma condition, levels of apoptosis and Δapoptosis were significantly higher than those observed for either 50% or 100% plasma (Fig 1A and 1B). Proliferation of cells irradiated, for the 0% plasma condition, was lower than that of cells irradiated in the presence of 50% or 100% plasma (Fig 1C), and a significantly higher proliferation inhibition percentage was observed for the 0% plasma (82%) versus the 100% plasma (61%) condition (Fig 1D). Quantification of 8-MOP concentration in the matrix revealed that for all 3 plasma concentrations a large amount of 8-MOP remained in the container after irradiation, and that the percentage of residual 8-MOP remaining in the irradiation container after ECP treatment was significantly higher for the 100% and 50% plasma conditions (94% in both cases) than the 0% plasma condition (79%) (Fig 1E).

### - Hematocrit: The limiting factor for *in vitro* ECP efficacy

**Fig 2:**
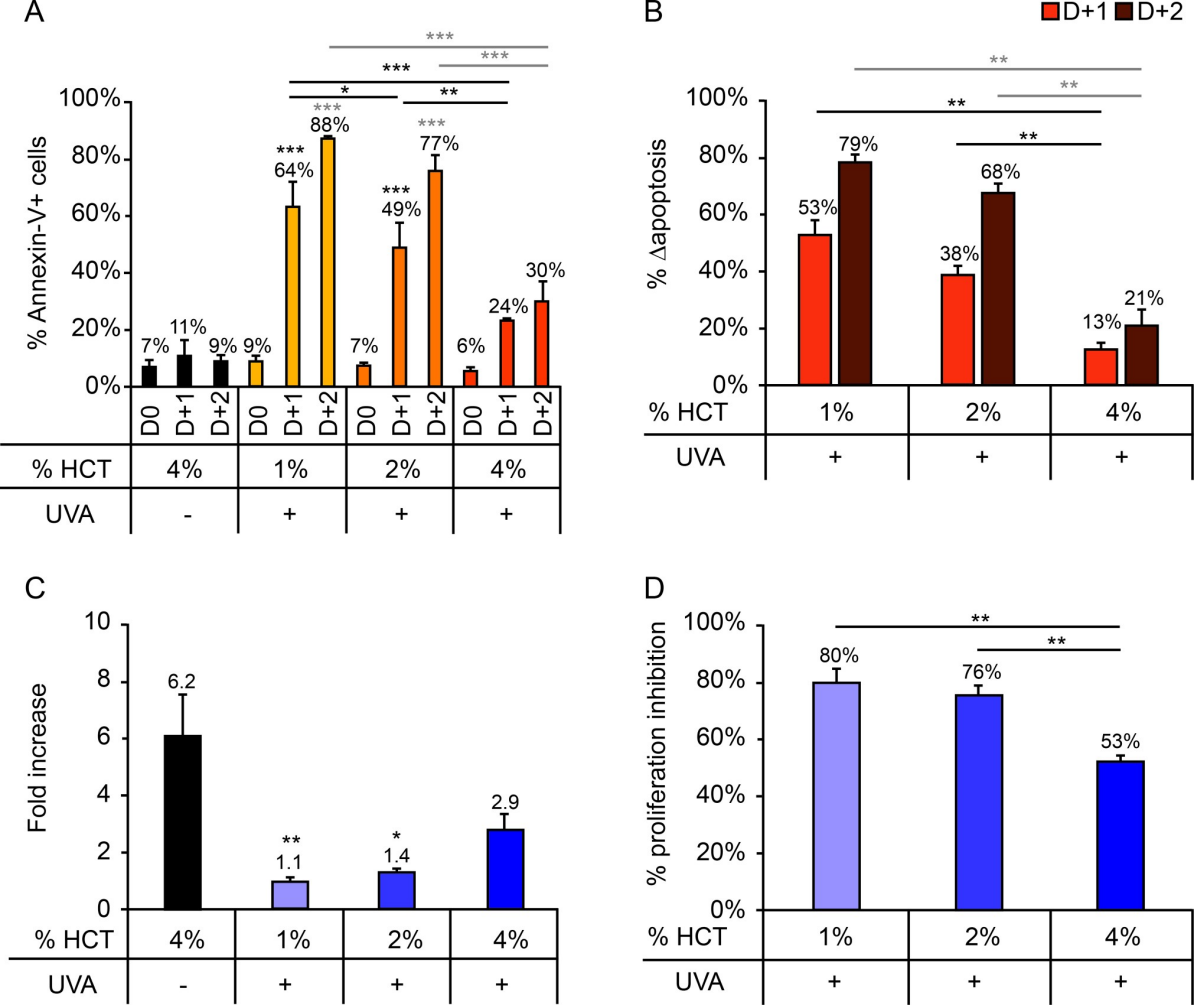
Impact of hematocrit (1%, 2%, or 4%) on cellular response after UVA irradiation (2 J/cm^2^). (A) Percentage of Annexin-V+ cells measured daily up to 2 days after irradiation. (B) Δapoptosis determined on days 1 and 2 post-irradiation. (C) Fold increase on day 3 post-irradiation relative to day 0. (D) Proliferation inhibition percentage, calculated as the percentage decrease in proliferation in irradiated versus control samples. For clarity, only statistically significant results are shown. *p<0.05; **p<0.01; ***p<0.001. Significant differences with respect to the control (UVA-) are indicated with an asterisk above the relevant bar, while comparisons between irradiated samples are indicated with an asterisk over a line linking the relevant bars. For Annexin-V+ cells (A) and Δapoptosis (B), comparisons performed on days 1 and 2 post-irradiation are indicated in black and gray, respectively.

Samples containing 3 different HCT were tested: 1%, 2%, and 4% (Fig 2). For all 3 conditions increases in cell death were observed 1 and 2 days after irradiation (Fig 2A). However, the level of apoptosis measured daily after irradiation decreased with increasing HCT concentration (Fig 2A). Δapoptosis was significantly lower in samples containing 4% HCT than those containing 1% or 2% HCT (Fig 2B). Interestingly, there was no significant difference in proliferation between control samples and those irradiated in the presence of 4% HCT (Fig 2C). The percentage proliferation inhibition in samples irradiated in the presence of 1% or 2% HCT was significantly higher than that of samples irradiated in the presence of 4% HCT (Fig 2D).

### - Cell density has no effect on the *in vitro* efficacy of ECP

**Fig 3:**
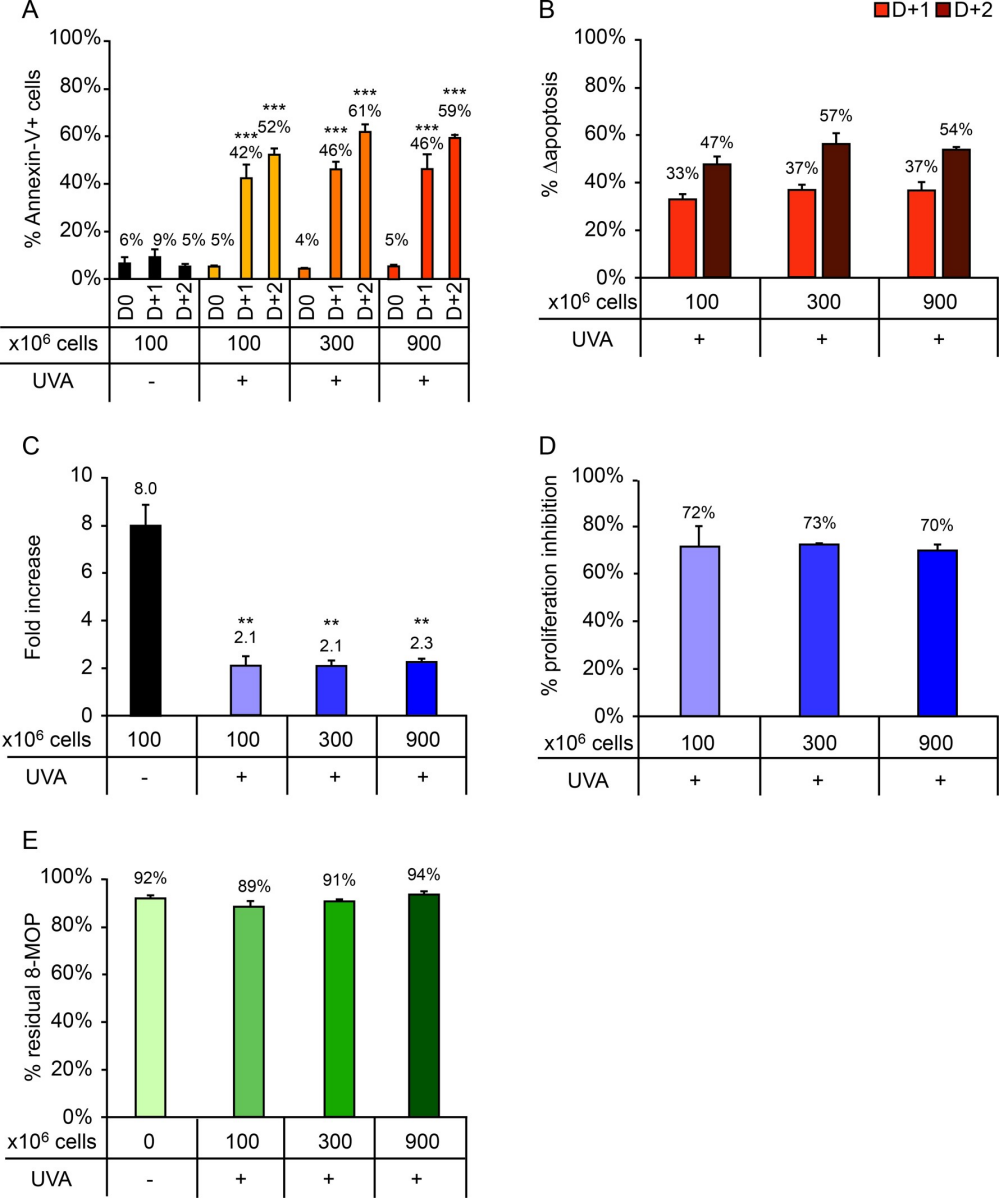
Impact of cell density (100 × 10^6^, 300 × 10^6^, and 900 × 10^6^ cells) on cellular response after UVA irradiation (2 J/cm^2^). (A) Percentage of Annexin-V+ cells measured daily up to 2 days after irradiation. (B) Δapoptosis determined on days 1 and 2 post-irradiation. (C) Fold increase on day 3 post-irradiation relative to day 0. (D) Proliferation inhibition percentage, calculated as the percentage decrease in proliferation in irradiated versus control samples. (E) Residual 8-MOP remaining in the irradiation container after treatment, expressed as a percentage of the pre-irradiation 8-MOP concentration. For clarity, only statistically significant results are shown. *p<0.05; **p<0.01; ***p<0.001. Significant differences with respect to the control (UVA -) are indicated with an asterisk above the relevant bar, while comparisons between irradiated samples are indicated with an asterisk over a line linking the relevant bars. For Annexin-V+ cells (A) and Δapoptosis (B), comparisons performed on days 1 and 2 post-irradiation are indicated in black and gray, respectively.

Samples containing 3 different cell densities were tested: 100 × 10^6^, 300 × 10^6^, and 900 × 10^6^ cells (Fig 3). For all 3 cell densities similar levels of cell death were observed 1 and 2 days post-irradiation (Fig 3A). Similarly, no significant differences in Δapoptosis (Fig 3B), fold increase (Fig 3C), or proliferation inhibition percentage (Fig 3D) were observed for the different cell densities. 8-MOP consumption was comparable for all 3 conditions (Fig 3E).

### - 8-MOP concentrations of 200 and 333 ng/ml result in comparable *in vitro* ECP efficacy

**Fig 4:**
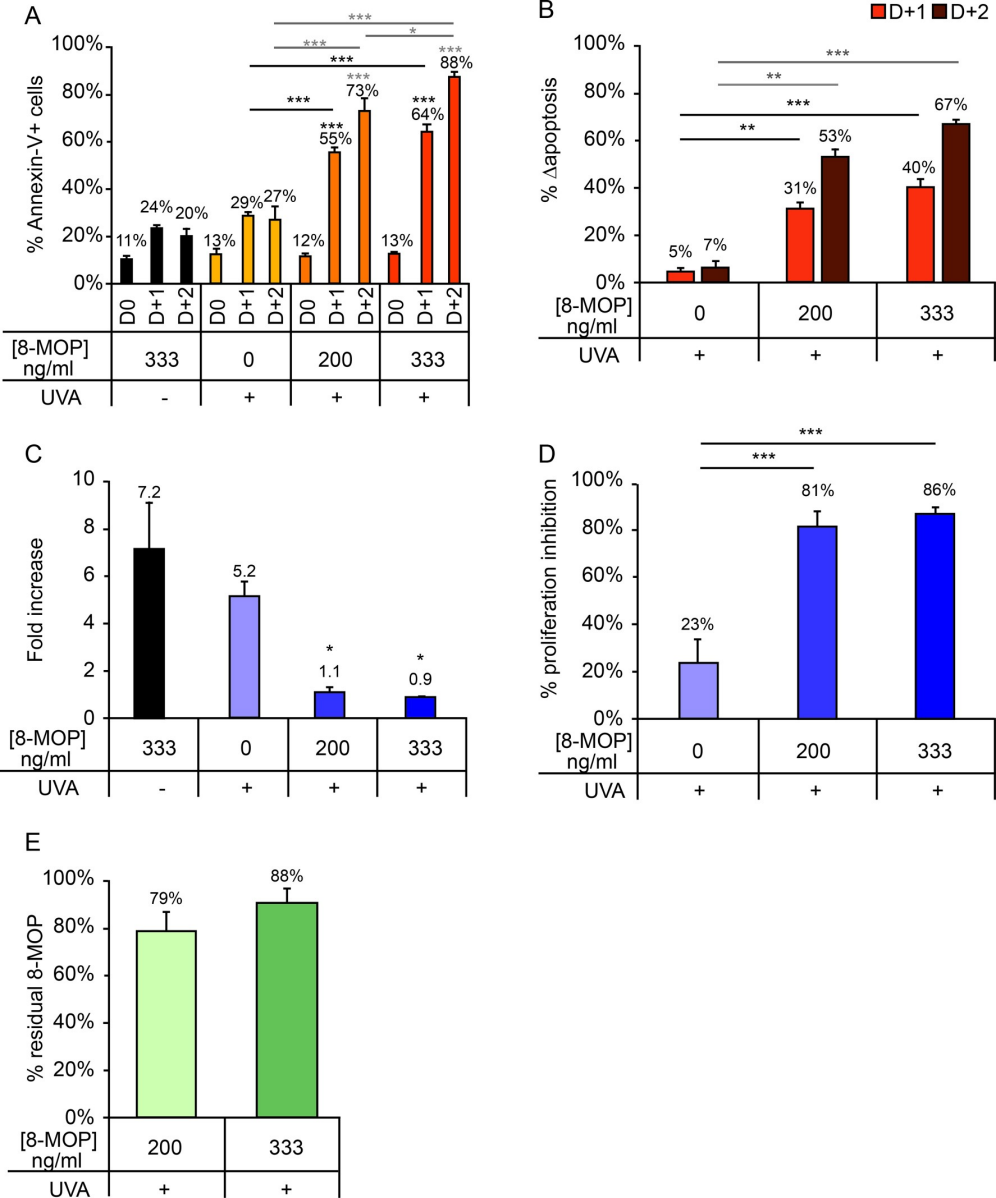
Impact of 8-MOP concentration (0 ng/ml, 200 ng/ml, or 300 ng/ml) on cellular response after a 2 J/cm^2^ UVA dose. (A) Percentage of Annexin-V+ cells measured daily up to 2 days after irradiation. (B) Δapoptosis determined on days 1 and 2 post-irradiation. (C) Fold increase on day 3 post-irradiation relative to day 0. (D) Proliferation inhibition percentage, calculated as the percentage decrease in proliferation in irradiated versus control samples. (E) Residual 8-MOP remaining in the irradiation container after treatment, expressed as a percentage of the pre-irradiation 8-MOP concentration. For clarity, only statistically significant results are shown.*p<0.05; **p<0.01; ***p<0.001. Significant differences with respect to the control (UVA -) are indicated with an asterisk above the relevant bar, while comparisons between irradiated samples are indicated with an asterisk over a line linking the relevant bars. For Annexin-V+ cells (A) and Δapoptosis (B), comparisons performed on days 1 and 2 post-irradiation are indicated in black and gray, respectively.

The effects of two 8-MOP concentrations (200 and 333 ng/ml) on *in vitro* ECP efficacy were compared with those observed for the sample lacking 8-MOP and the non-irradiated control (Fig 4). Irradiation in the absence of 8-MOP resulted in no significant increase in cell apoptosis with respect to the non-irradiated control, while the addition of 8-MOP significantly increased apoptosis (Fig 4A). Δapoptosis did not differ significantly between the samples containing 200 and 333 ng/ml 8-MOP (Fig 4B). Similarly, fold increase of T-cells irradiated in the absence of 8-MOP was comparable to that observed for the control condition (Fig 4C). No differences in proliferation inhibition percentage were observed between the 200 ng/ml and 333 ng/ml conditions (Fig 4D), nor was there any significant difference in the percentage of residual 8-MOP between the 200 ng/ml (79%) and 333 ng/ml (88%) conditions. For both conditions, 8-MOP consumption during treatment corresponded to approximately 12 × 10^3^ ng (Fig 4E).

### - Increasing the UVA dose can counteract the limiting effect of a high hematocrit concentration on the cellular response

**Fig 5:**
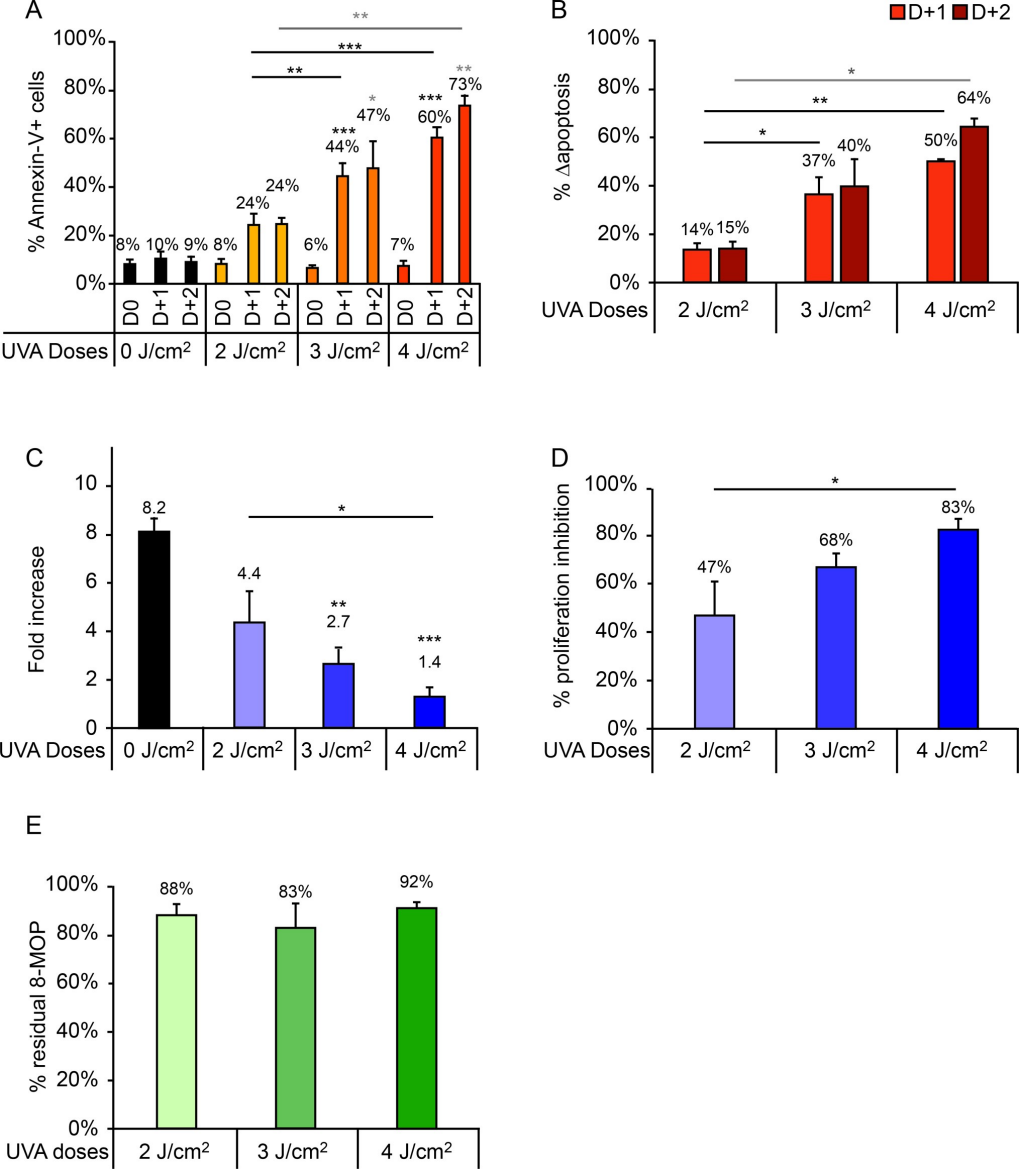
Impact of UVA dose (2 J/cm^2^, 3 J/cm^2^, and 4 J/cm^2^) on cellular response of samples treated with 4% HCT in the irradiation bag. (A) Percentage of Annexin-V+ cells measured daily up to 2 days after irradiation. (B) Δapoptosis determined on days 1 and 2 post-irradiation. (C) Fold increase on day 3 post-irradiation relative to day 0. (D) Proliferation inhibition percentage, calculated as the percentage decrease in proliferation in irradiated versus control samples. A pairwise comparison of each treatment condition was performed. For clarity, only statistically significant results are shown: *p<0.05; **p<0.01; ***p<0.001. Significant differences with respect to the control (UVA -) are indicated with an asterisk above the relevant bar, while comparisons between irradiated samples are indicated with an asterisk over a line linking the relevant bars. For Annexin-V+ cells (A) and Δapoptosis (B), comparisons performed on days 1 and 2 post-irradiation are indicated in black and gray, respectively.

As described above, a decrease in cellular response was observed in cell preparations containing more than 2% HCT (Fig 2). Next, we investigated whether this limiting effect could be overcome by increasing the UVA dose. We examined the effects of 3 different doses in the presence of 4% HCT: 2, 3, and 4 J/cm^2^ (Fig 5). The higher the UVA dose, the greater the level of apoptosis (Fig 5A). Doses of 3 and 4 J/cm^2^ resulted in significantly higher Δapoptosis at 1 day post-irradiation than the 2 J/cm^2^ dose (Fig 5B). Consequently, the level of proliferation of cells irradiated with 3 and 4 J/cm^2^ was lower than that of cells irradiated with 2 J/cm^2^ (Fig 5C). The proliferation inhibition percentage of cells irradiated with 4 J/cm^2^ was significantly higher than that of cells irradiated with 2 J/cm^2^ (Fig 5D). Finally, no differences in the percentage of residual 8-MOP in the irradiation container was observed across UVA doses (Fig 5E).

## Discussion

In this study, we present a standardized methodical approach for the *in vitro* assessment of ECP, and use this approach to quantify for the first time the influence of individual components of the cell preparation and of UVA dose on *in vitro* ECP efficacy. Experiments were performed using the immortalized JURKAT cell line, thereby avoiding the response to treatment variability and mitogen-induced proliferation efficacy associated with PBMCs obtained from different donors, and allowing standardization of the treatment to ensure a more precise analysis. Apoptosis of JURKAT cells increased in a time-dependent manner over a 2-day period after UVA irradiation, irrespective of variations in the composition of the cell preparation in the irradiation container. This apoptotic process may account for the progressive immunomodulatory effects observed in patients after ECP treatment [7, 40].

**Fig 6:**
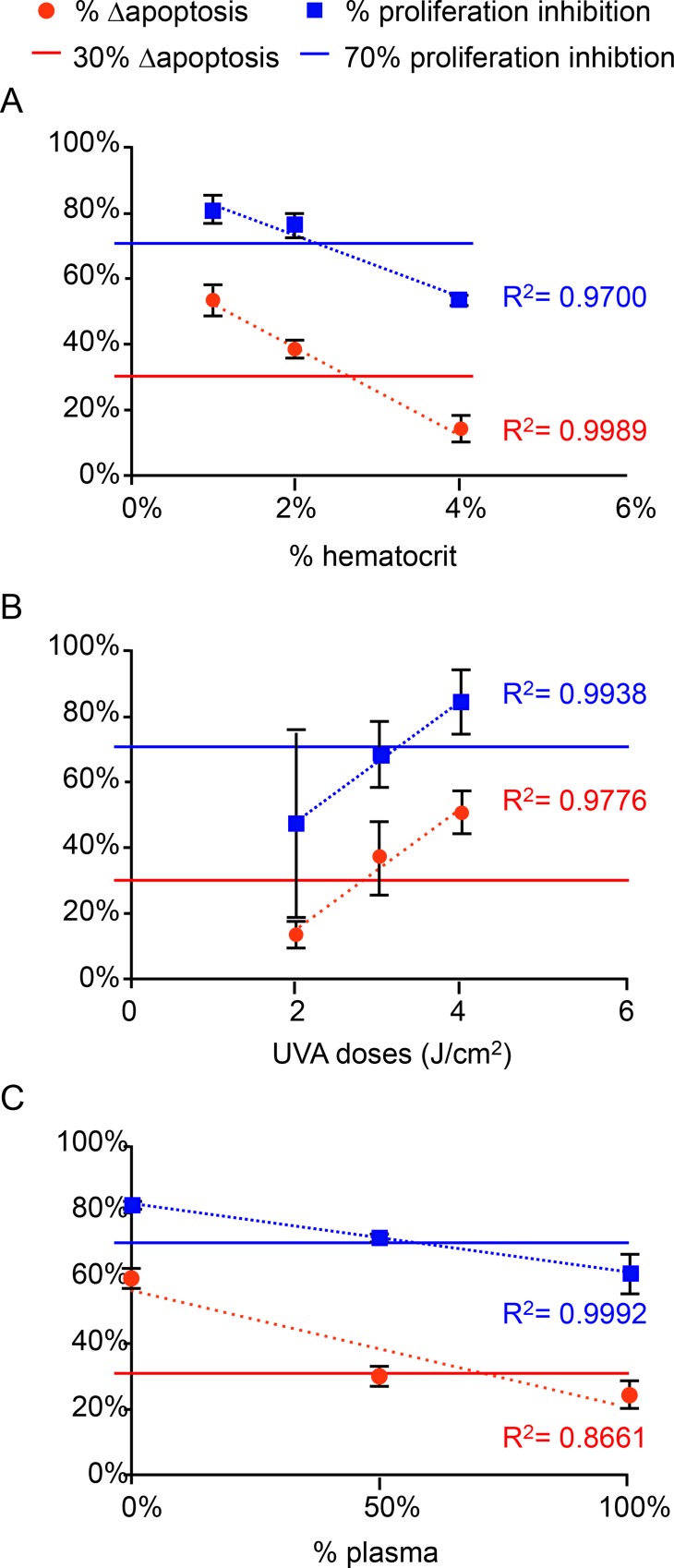
Comparison of percentages of proliferation inhibition and Δapoptosis with corresponding acceptance criteria. Linear correlation of Δapoptosis on day 1 (dotted red line) and proliferation inhibition percentage on day 3 (dotted blue line) for (A) increasing hematocrit (% HCT), (B) UVA dose, and (C) plasma percentage. Evaluation criteria are indicated with solid lines: blue, 70% proliferation inhibition at day 3 [39]; red, 30% Δapoptosis at day 1 [27].

To standardize our method we used fixed component ratios for the mixture in the irradiation container and defined baseline settings for the irradiation device. To investigate potential dose effects of alterations in plasma, HCT, and 8-MOP concentration, as well as T-cell density and UVA dose, we applied a one-variable-at-a-time approach. Experiments revealed a dose effect on Δapoptosis and proliferation inhibition percentage for 3 parameters: HCT, plasma ratio and UVA dose. Moreover, for each of these 3 parameters we observed a linear correlation between Δapoptosis and proliferation inhibition percentage, with a coefficient of determination close to or >0.9 (Fig 6A–6C, dotted lines). As expected, HCT was the most stringent component, likely owing to its high absorbance in the UVA range and its shielding effect on other cells [13].

We assessed our findings by applying 2 evaluation criteria previously used as quality-control benchmarks to validate ECP treatments by Jacob *et al*. [39] and Taverna *et al*. [27], respectively: (i) 70% proliferation inhibition after 3 days in culture; and (ii) 15% Δapoptosis after 1 day in culture. It should be noted that these evaluation criteria were both established in studies using PBMCs from patients with GvHD or CTCL [27, 39]. Importantly, under our standardized ECP conditions using JURKAT cells these criteria were fulfilled in most cases. We used these criteria to assess the impact on *in vitro* ECP efficacy of alterations in each of the aforementioned ECP parameters. Our findings demonstrate a correlation between Δapoptosis on day 1 post-irradiation and proliferation inhibition on day 3 post-irradiation, thereby allowing prediction of the outcome of the experiment as early as 1 day after cellular treatment. Specifically, in our experimental settings the threshold of 70% proliferation inhibition on day 3 was reached when Δapoptosis on day 1 exceeded 30% (Fig 6A–6C, solid blue and red lines, respectively). Based on this correlation, we found that HCT concentration should be maintained at <2.6% when using a UVA dose of 2 J/cm^2^ (Fig 6A), while a UVA dose of at least 3 J/cm^2^ is required when using HCT of 4% (Fig 6B). Similar outcomes were observed using 4% HCT and 3 J/cm^2^ UVA as for 2% HCT and 2 J/cm^2^ UVA (Fig 6A and 6B). According to Brosig *et al*. (2016), the HCT in the irradiation container should rarely reach 4%, since the mean percentage of HCT in a 200-ml leukapheresis extract ranges from 1.3–3.7% before dilution, depending on the apheresis machine used [19]. The off-line ECP technique used in the present study allows quality control steps to be performed by measuring the HCT before irradiation [27, 39]. Based on the recorded HCT, the UVA dose can be adjusted between 1 and 3 J/cm^2^ [41].

Plasma ratio also had an impact, albeit less pronounced than that of HCT, on the cellular response after ECP treatment (Fig 6C). We observed a greater cellular response, in terms of apoptosis induction and proliferation inhibition, when using a 0% plasma condition than that observed for the 100% plasma matrix (Fig 6C), suggesting that leukapheresis products should ideally be diluted in saline solution rather than plasma. We conducted experiments using a pool of plasma in order to minimize inter-donor variability [42]. How the biochemical characteristics of the plasma of individual patients affects ECP outcomes will be an interesting axis of research for future studies. By selecting plasma from donors based to their lipid balance, the impact of triglyceride levels on ECP outcomes could also be studied. Moreover, our model for the *in vitro* assessment of ECP efficacy could be used to determine the most appropriate moment for leukapheresis processing (i.e., pre- or post-prandial).

Measurement of the percentage of residual 8-MOP remaining after irradiation further highlighted the impact of plasma concentration on ECP. The presence of plasma prevented the adsorption of 8-MOP on plastic, as evidenced by the lower percentage of residual 8-MOP observed in the absence of plasma. Our results are in line with those of Hähnel *et al*. and confirm that 8-MOP can interact with plasma proteins [23]. Moreover, large amounts of 8-MOP remained in the container after irradiation when using starting doses of 200 or 333 ng/ml 8-MOP. We also observed no effect of alterations in cell density on ECP efficacy and 8-MOP consumption, indicating an excess of 8-MOP molecules relative to the amount of cells studied. For both of the 8-MOP concentrations tested our evaluation criteria were fulfilled and comparable ECP efficacy was observed, indicating that both concentrations were sufficient to ensure passive diffusion of 8-MOP into cells to induce apoptosis [43]. In our experimental conditions, no significant differences were highlighted between both 8-MOP concentrations despite the slight increase in apoptosis noticed when using 5 ml of 8-MOP compared to 3ml.

Our findings show that the cellular response to ECP treatment can be standardized by restricting the proportion of certain blood components, including HCT and plasma, to a specific range. Most currently available apheresis machines allow standardization of the leukapheresis product in a constant volume of saline with low non variable levels of HCT [19]. Further studies will be required to investigate the correlation between the in vitro cellular response to ECP (using standardized evaluation criteria) and clinical outcome using the methodology presented here, particularly in patients with different pathologies (GvHD, CTCL).

Our standardized method could also be used as a benchmarking tool, for example to test and compare the *in vitro* ECP efficacy of innovative cellular preparation methods, such as mini buffy coat ECP [30, 44] or cryo-ECP [45, 46], before use in a clinical setting. The use of mini buffy coat preparations involves very low dilutions of the buffy coat, and consequently high concentrations of HCT and plasma and a lower irradiated volume. Applying the 2 evaluation criteria described here, our methodology could be used to determine the effect on *in vitro* ECP efficacy of the reduced volume and more absorbent matrices of mini buffy coat preparations. Conversely, in the case of cryo-ECP, in which cells are irradiated in the presence of very low HCT and plasma ratio, our method could be used to determine the influence of the matrix components on the cellular response and to optimize the UV dose. Ultimately, such a benchmarking tool would allow for adjustment of the system settings and would produce in vitro cellular responses similar to those seen in the standard ECP procedure, irrespective of the initial cellular preparation method used. This would ensure that patients are reinfused with an optimized cellular preparation.

In conclusion, our standardized method for the *in vitro* assessment of ECP efficacy highlights the key limiting factors associated with this technique, and underscores the influence of the relative proportions of the different components of the cell preparation in the irradiation container. The off-line system used here is versatile and can be adapted to different patients, diseases, and preparation methods in order to evaluate and compare cellular treatment efficacy. Taken together, our findings constitute an important step forward towards the development of a standardized method to evaluate ECP efficacy.
